# Population dynamics of a natural red deer population over 200 years detected via substantial changes of genetic variation

**DOI:** 10.1002/ece3.2063

**Published:** 2016-04-05

**Authors:** Gunther Sebastian Hoffmann, Jes Johannesen, Eva Maria Griebeler

**Affiliations:** ^1^ Department of EcologyInstitute of ZoologyJohannes Gutenberg‐University of MainzD‐55099MainzGermany

**Keywords:** Cervidae, *Cervus elaphus*, microsatellites, mtDNA, natural history collections

## Abstract

Most large mammals have constantly been exposed to anthropogenic influence over decades or even centuries. Because of their long generation times and lack of sampling material, inferences of past population genetic dynamics, including anthropogenic impacts, have only relied on the analysis of the structure of extant populations. Here, we investigate for the first time the change in the genetic constitution of a natural red deer population over two centuries, using up to 200‐year‐old antlers (30 generations) stored in trophy collections. To the best of our knowledge, this is the oldest DNA source ever used for microsatellite population genetic analyses. We demonstrate that government policy and hunting laws may have strong impacts on populations that can lead to unexpectedly rapid changes in the genetic constitution of a large mammal population. A high ancestral individual polymorphism seen in an outbreeding population (1813–1861) was strongly reduced in descendants (1923–1940) during the mid‐19th and early 20th century by genetic bottlenecks. Today (2011), individual polymorphism and variance among individuals is increasing in a constant‐sized (managed) population. Differentiation was high among periods (*F*_ST_ > ***); consequently, assignment tests assigned individuals to their own period with >85% probability. In contrast to the high variance observed at nuclear microsatellite loci, mtDNA (D‐loop) was monomorphic through time, suggesting that male immigration dominates the genetic evolution in this population.

## Introduction

Evolution is change in gene pools over time. While long‐term evolutionary processes have been studied by analyzing temporal patterns in fossil records (Wandeler et al. 2007) and footprints of selection (Hansen et al. 2010; Gautier and Naves 2011), the study of micro‐evolutionary processes has often been limited to organisms with short generation times of at most a few weeks (Balanya et al. 2006). With the advent of molecular genetic approaches, natural history collections (NHC) have become a new important source of tissue material for population genetic studies, opening up for studies comparing historical and contemporary natural populations of species with long generation times of several years (Wandeler et al. 2007). Using historical samples, anthropogenic influence on the gene pool of populations has been documented for migration (gene flow) in the African wild dog (Roy et al. 1994), on population sizes in Greater prairie chicken (Bouzat et al. 1998) and introgression in fish populations due to stocking (Ciborowski et al. 2007). Although NHC offer various possibilities for retrospective analyses of evolution, there are still limitations. Besides DNA degradation through time, the availability of museum samples from a specific geographical region is often scarce, but essential when assessing genetic and demographic impacts on specific populations.

Like most other large mammal species, European red deer (*Cervus elaphus* L.) has for decades or even centuries been exposed to anthropogenic pressures, affecting its genetic structure (Hartl et al. 2003). As an important game animal and meat supplier, population sizes have fluctuated highly depending on government policies, hunting laws, and translocations (Fernandez‐Garcia et al. 2014). In recent decades, rigorous hunting schedules have favored large, branched antlers by the protection of these individuals from harvesting until they could reproduce several times (Hartl et al. 2003), likely causing strong selection pressure on this trait (Hartl et al. 1991, 1995). Additionally, significant damages to forestry and agricultural plants have initiated large‐scale eradication of red deer populations. Several population genetic studies have shown significant differentiation among artificially separated red deer populations (Hartl et al. 1990; Schreiber et al. 1994), inbreeding depression in isolated populations (Zachos et al. 2007), and have found evidence for translocations (Frantz et al. 2006). All studies conducted so far on red deer have examined extant populations using muscle tissue (Coulson et al. 1998; Zachos and Hartl 2011), thus making retrospective inferences on evolutionary processes.

In this study, we use DNA from up to 200‐year‐old antlers from a trophy collection to analyze the temporal evolution of a confined red deer (*C. elaphus* L.) population. All samples derive from a small geographical area (<10 km^2^), which is easily daily covered by red deer, thus representing a locality‐based population. We analyzed the change in the genetic constitution of this population over time by sampling antlers from three periods (1813–1861, 1923–1940, 2011), and evaluating ten microsatellite loci. To detect possible translocations of foreign female individuals into the population, we additionally sequenced an 800‐bp fragment of the highly variable mitochondrial control region. Microsatellite loci commonly used in genetic studies utilize DNA extracted from muscle tissue (Kuehn et al. 2004) and have successfully been applied to old antler tissue (Hoffmann and Griebeler 2013). Our study reveals dramatic changes in the population's gene pool over time for microsatellite loci, but none for the control region. To the best of our knowledge, our study is based on the oldest microsatellites ever used for a population study.

## Materials and Methods

### Study population

A total of 31 individuals were genotyped through time in a population at Neuwied, Germany (Table S1). The individuals originated from three periods: 1813–1861 (*N* = 6), 1923–1940 (*N* = 8), and 2011 (*N* = 17). In the following text, we refer to these three subsamples as the Old, Middle‐aged, and Young population, respectively. As the samples in population Old and Middle‐aged originate from a time period, these two populations represent the average of genetic diversity during these periods rather than instant populations.

### Population history

In the period 1813–1861, the Neuwied population was large. Although the samples were derived from the private hunting area of the princes of Neuwied from an area of only 10 km^2^, there were other red deer populations around with few barriers to migration and a low degree of fragmentation in an overall continuous habitat. During the German revolution years 1848–1849, no hunting laws were in action. This short period led to massive reductions in red deer population sizes and large‐scale extinctions of red deer populations due to poaching and unregulated harvesting. During the second half of the 19th century, hunting laws were reenacted which allowed populations to re‐establish in many areas (Kuehn et al. 2003). After re‐establishment, population sizes were reduced again due to increasing damages to agriculture, but also poaching during and after World War 1. Our second population samples come from a period representing the end phase of overexploitation (1923–1940). Following the 1950s, red deer populations expanded into many forested areas. For the first time, rigorous hunting schedules favoring large, branched antlers were introduced (Hartl et al. 2003). This schedule mostly spares males with large antlers from being hunted during their reproductive years. Although migration into the population from other areas is possible, the contemporary Neuwied population is more fragmented than in former times. It is confined in the south by the river Rhine, and in the west and east, it is fenced in by motorways and highways. Migration and gene flow is presumably highly restricted in this population (further information about population history and study area in Appendix S1 in Supporting Information).

### DNA preparation

The DNA of individuals from the Old (*N* = 6) and Middle‐aged populations (*N* = 8) was extracted from a small borehole drilled on the backside of antlers, following the method of Hoffmann and Griebeler (2013). As only males produce antlers in red deer, all sampled individuals were males. The samples of population Young were randomly taken from hunted individuals (*N* = 17). DNA was extracted from muscle tissue from both sexes (11 females, six males) with the “High pure PCR template preparation Kit” (Roche Diagnostics) using the standard protocol recommended by the manufacturer. All DNA samples were stored at −20°C. The DNA content of every sample was measured with NanoDrop1000 (Peqlab, Erlangen, Germany). The DNA for PCR was diluted in elution buffer to a final concentration of 50–100 *μ*g/mL.

### Microsatellite laboratory procedure

We analyzed 10 microsatellite loci with dinucleotide repeat motifs: Ilsts06, CSPS115, MM12, CSSM16, Haut14, Inra35, CSSM22, CSSM19, CSSM14, BM1818 (Kuehn et al. 2004). All primers were developed for cattle (*Bos primigenius tauris*), but were identified by Kuehn et al. (2004) as applicable to red deer. DNA extracted from antler and muscle tissue was treated identically. The loci were amplified in two multiplex reactions (Table S2) using the Qiagen Multiplex Kit (Qiagen, Basel, Switzerland). The reaction volume for each sample consisted of 5 *μ*L Qiagen Multiplex PCR Mastermix (contains dNTPs, Qiagen HotStar Taq DNA Polymerase and 6 mmol/L MgCl2), 1 *μ*L Primer Mix (2 *μ*mol/L of each Primer), 2.5 *μ*L RNase‐free water, and 1.5 *μ*L DNA; the final reaction volume was 10 *μ*L for each DNA sample. PCRs were performed on a PerkinElmer GeneAmp 9600 thermocycler (Applied Biosystems, Foster City, CA) using the recommended multiplex PCR protocol (Qiagen) with the following cycling conditions: 15 min at 95°C for initial denaturation; then 40 cycles of 40 sec at 94°C, 90 sec at 57°C, and 90 sec at 72°C, followed by 30 min at 60°C, and a final hold at 4°C.

Purified microsatellite PCR fragments were run on a 3130xl capillary sequencer (Applied Biosystems) using a GS500 ROX size standard. The genotypes were analyzed with GENEMAPPER, version 4.0 (Applied Biosystems). To avoid false allele scoring, we only scored alleles with fluorescent values above 3000 and a distinctive, characteristic form at each locus. Samples with ambiguous peaks were assessed as no amplification success. In total, 60% of all individuals were genotyped twice, after independent PCRs, to ensure reproducibility of genotypes. Null alleles, large allele dropouts, or scoring errors due to stuttering were checked with the MicroChecker software (Version 2.2.3) (Van Oosterhout et al. 2004).

### MtDNA laboratory procedure

To examine female‐based genetic structure, we sequenced the highly variable mtDNA control region (D‐loop). The control region has previously been used in a biogeographical study of red deer in Europe to assign foreign haplotypes (Niedzialkowska et al. 2011). The control region was first amplified with self‐made primers (Table S3), which amplify the sequence between Pro‐tRNA and Phe‐tRNA (800 base pairs). In case of partial amplification success, we re‐sequenced the control region in stepwise amplifications with newly designed specific forward and reverse primers (Table S3).

Each 25‐*μ*L PCR consisted of 21 *μ*L of H_2_O, 1 *μ*L of each respective primer, and 2 *μ*L of DNA extract contained in a 0.5‐mL PCR tube with illustra™ puReTaq Ready‐To‐Go PCR Beads (GE Healthcare, Buckinghamshire, United Kingdom). The final volume of 25 *μ*L contained 200 *μ*mol/L of each dNTP, 10 mmol/L Tris–HCL (pH 9.0, at room temperature), 50 mmol/L KCl, 1.5 mmol/L MgCl_2_, approximately 2.5 units of puReTaq DNA polymerase, reaction buffer and BSA, dATP, dCTP, dGTP, and dTTP. Thermocycling started at 95°C for 5 min, followed by 40 cycles of 94°C for 75 sec, then by 60°C for 90 sec, and then by an extension at 75°C for 75 sec. After a final extension at 72°C for 7 min, the reaction was stored at 4°C. Reactions were performed on a TGradient Biometra Thermocycler (Biometra, Göttingen, Germany). PCR products were separated using gel electrophoresis (1% Agarose) and fragments controlled for expected size and band quality. The amplified PCR fragments were purified using the Roche High Pure PCR Product Purification Kit (Roche Diagnostics).

MtDNA amplification products were sequenced in both directions with BigDye chemistries on a 3130xl capillary sequencer (Applied Biosystems). Sequence data were aligned and edited with BioEdit (Version 7.1.3) (Hall 1999).

### Population genetics analyses of microsatellites

The genetic analyses were based on three genetic indices providing assessments of within‐population dynamics: (1) genetic diversity – including the assessment of the magnitude of genetic drift, (2) inbreeding estimates, and (3) variance of heterozygosity among individuals within periods. While a reduction in genetic diversity is indicative of genetic drift and bottlenecks, estimates of inbreeding are more difficult to assess. Assuming no selection on heterozygotes, negative *F*_is_ values may be caused by mating success of foreign individuals or when the individuals who make up the population sample are siblings (Rasmussen 1979; Johannesen and Lubin 1999), while positive *F*_is_ values implicate any sort of Wahlund effect (Johnson and Black 1984), which is a deficiency of heterozygotes compared to Hardy–Weinberg expectations. Variance differences in individual heterozygosity give implications for the stability of the population/breeding system. Low variance is evidence for even breeding success and ancestry, whereas high variance indicates perturbations from equilibrium. The magnitude of change should be manifested in the differentiation among time samples.

We first tested for linkage disequilibrium in time samples to assure independency among microsatellite loci [GenePop on the web, Version 4.2 (Rousset 2008)]. Because of different sample sizes in the three periods, we do not calculate absolute number of alleles per locus, but effective number of alleles in relation to sample size (allelic richness). The effective number of alleles per locus and effective number of private alleles per sample were calculated with HpRare (Version 1.1) (Kalinowski 2005) applying the Rarefaction method. Expected and observed heterozygosities, as well as inbreeding estimates *F*_is_ and *F*_st_, as a measure of variation of allele frequencies, were calculated with Arlequin (Version 3.5.1.2) (Excoffier et al. 2005). To assess the likelihood that an individual belongs to a particular time sample, we used the Bayesian assignment method as implemented in GeneClass (Version 2.0) (Piry et al. 2004). In this test, we excluded each individual to be assigned from the reference population. The magnitude of genetic drift was estimated with BottleSim (Kuo and Janzen 2003) (assumed parameters: random mating, longevity of organism 14 years, sexual maturity at age of 4, population size prebottleneck: 100, postbottleneck: 40, sex ratio: 1:1, number of iterations: 50) and compared to the observed data. In this analysis, we assessed the level of genetic drift expected in samples Middle‐aged and Young based on the observed heterozygosity of the respective previous sample. We estimated two drift scenarios: (1) drift based on heterozygosity in the original population (1813–1861) and (2) drift based on observed heterozygosity in the second sampling period (1923–1940). The model parameter generation time was set as the midpoint of the respective periods. Finally, *t*‐tests (Excel 2010) for samples with homogeneity and also for inhomogeneity of variance as implemented in Excel 2010 were applied to test for differences between heterozygote loci/individual among periods. *F*‐tests of inhomogeneity of variance between samples were also conducted with Excel 2010.

### MtDNA analysis

The mtDNA control region sequences were aligned with Mega (Version 5.1) (Tamura et al. 2011). Because only a single haplotype was found (see Results), no timescale analysis was performed. Nevertheless, we compared this haplotype to those found in other geographical regions of Europe in a GenBank search.

## Results

### DNA extraction and quality

High‐quality DNA was extracted from all antlers in moderate‐to‐high concentrations (44–370 *μ*g/mL). Both microsatellites and mtDNA amplified unambiguous peaks with high fluorescent values. Microsatellite amplification success rate was 97.9%. DNA quality was verified by genotyping 60% of all individuals twice, which yielded 100% concordance (for a complete table of microsatellite genotypes see Table S4). The test for null alleles revealed no evidence for null alleles, large dropouts, or false alleles due to stuttering (MicroChecker 2.2.3).

### Linkage disequilibrium

The tests for pairwise linkage disequilibrium between microsatellite loci revealed three significant values (*P* < 0.05) of 135 comparisons. This frequency is lower than expected by chance (5% level). Thus, these results indicate independent inheritance of the studied loci.

### Temporal population genetic indices

**Figure 1:**
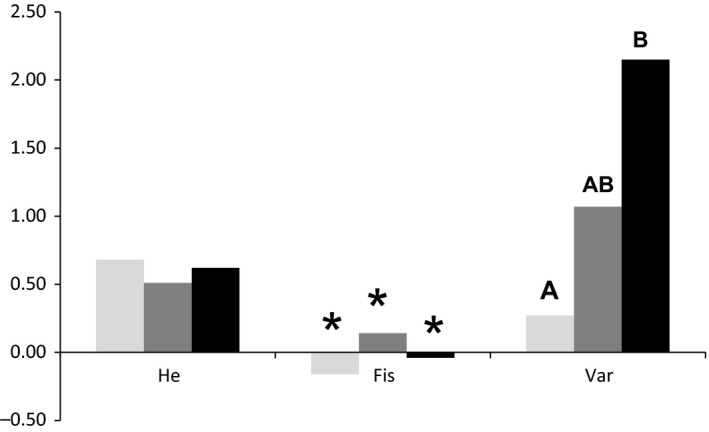
Summary of relative estimates of diversity (expected heterozygosity of the time sample, *H*_e_), inbreeding coefficient of the time sample (*F*_is_) and variance among individuals within samples in number of heterozygote loci (Var). Stars (*) indicate estimates significantly different from zero. Different letters show significant differences in the variance in number of heterozygote loci/individual based on 10 microsatellite loci. Different shades of gray mean year of sampling, light gray = 1837, gray = 1930, black = 2011.

When correcting for differences in sample sizes, population Old had the highest effective number of alleles (3.99), followed by population Young (3.49), whereas population Middle‐aged had the lowest number (2.71). Consistent with the differences in effective allele numbers, the expected heterozygosity was similar in population Old (*H*_e_ = 0.68) and young (*H*_e_ = 0.60), but lower in population Middle‐aged (*H*_e_ = 0.51). Inbreeding estimates *F*_is_ were significantly positive in the population Middle‐aged (0.148), whereas they were significantly negative in population Young (−0.039) and Old (−0.158) (Fig. 1).

The number of heterozygote loci per individual differed significantly between all pairwise comparisons of population samples (periods) (Old vs. Middle‐aged: df = 12, *t* value = −6.295, *P* < 10^−4^; Middle‐aged vs. Young: df = 23, *t* value = 2.463, *P* = 0.011; Old vs. Young: df = 21, *t* value = −3.602, *P* < 0.001). The average number of heterozygote loci per individual was 7.67 ± 0.52 SD in population Old, 4.75 ± 1.04 SD in population Middle‐aged, and 6.18 ± 1.47 in population Young (Table 1). Variance in individual (i.e., observed) heterozygosity per period increased significantly through time. Variance inhomogeneity was only significant between population Old and Young (df = 16, *F* = 8.08, *P* = 0.015; Old vs. Middle‐aged: df = 7, *F* = 4.018, *P* = 0.072; Middle‐aged vs. Young: df = 16, *F* = 2.011, *P* = 0.177).

**Table 1:** Genetic diversity estimates for three time‐period populations. Number of sampled animals (*N*), absolute number of alleles per locus (*n*_a_), effective number of alleles per locus (*n*_e_), effective number of private alleles per population (*p*_e_), expected (*H*_e_) and observed (*H*_o_) heterozygosities in studied populations over all loci, mean number of heterozygote loci/individual (N het), and variance of heterozygote loci/individual (Var)

Population	*N*	*n*_a_	*n*_e_	*p*_e_	*H*_e_	*H*_o_	N het	Var
Young	17	4.6	3.49	0.67	0.60	0.62	6.18	2.15
Middle‐aged	8	3.0	2.71	0.27	0.51	0.42	4.75	1.07
Old	6	4.2	3.99	1.11	0.68	0.78	7.67	0.27

### Genetic drift simulation

**Figure 2:**
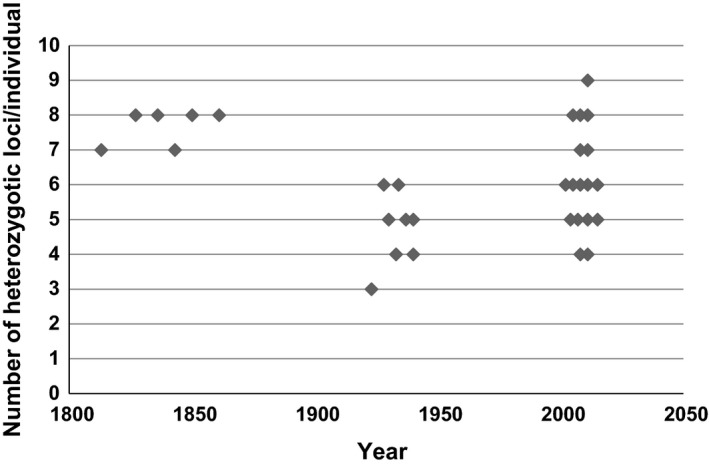
Number of heterozygotic loci per individual in the different time periods/years. Although all individuals were sampled in 2011, the multiyear presentation of the young sample is chosen for convenience to demonstrate variability between individuals.

In the bottlenecked Middle‐aged population, we detected shifts in allele frequencies at most loci (Table S5). As seen in Figure 2, the overall proportion of heterozygote loci increased in population Young, but not in each of the individuals.

**Figure 3:**
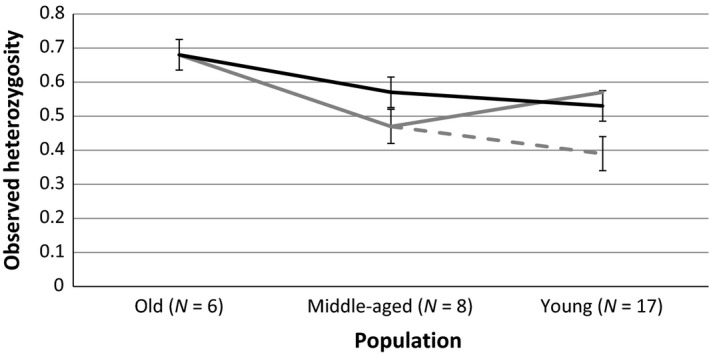
Observed heterozygosity of the time samples and genetic drift simulated in BottleSim (Kuo and Janzen 2003) (gray full line = observed heterozygosity, black full line = simulated heterozygosity prebottleneck, dotted line = simulated heterozygosity postbottleneck, error bars = standard error).

Genetic diversity in Middle‐aged is reduced and lower than expected through drift based on population Old. In contrast, diversity is higher in population Young than predicted by drift based on population Middle‐aged (Fig. 3).

### Assignment and genetic differentiation among periods

Genetic differentiation among populations from the three periods was significant with moderate‐to‐high *F*_st_ values (population Young vs. Old: *F*_st_ = 0.06; Middle‐aged vs. Young: *F*_st_ =0.11; Middle‐aged vs. Old: *F*_st_ = 0.09). The assignment test conducted with multilocus microsatellite genotypes assigned each individual from a time sample with a probability > 0.84 to this sample.

### MtDNA analysis

All 31 individuals sampled shared an identical mtDNA haplotype (656 bp, GenBank accession number: KU577263), making a detection of introgression of foreign haplotypes into the population impossible. The haplotype is new; that is, it is not recorded in GenBank (assessed 05.01.2016). The most similar haplotype found in GenBank was J729E with 99% similarity (655 of 656 bp).

## Discussion

Inferences of anthropogenic impacts on natural populations of mammals with long generation times have so far relied on retrospective analyses of contemporary populations. In this study, we analyzed the genetic evolution of a single red deer population sampled at three periods over two centuries. Assuming a generation time (mean distance between two generations) of 7 years (Coulson et al. 1998), successive population samples were separated by 11 up to 17 generations. During this time, the population experienced substantial genetic changes at the ten nuclear microsatellite loci analyzed, while sequence variation in the normally, highly variable mitochondrial D‐loop was absent. The historically high nuclear genetic diversity was followed by a diversity decrease and a subsequent diversity increase. This pattern is consistent with our understanding of hunting pressures during the two centuries (see Population history in Material and Methods).

Inferences of population dynamics were obtained by time‐based analyses, which provide three basic indices for assessing within‐population dynamics: (1) genetic diversity (*H*_e_), (2) inbreeding estimates (*F*_is_), and (3) variance in individual heterozygosity within periods. The relative levels of these indices among the three samples provide evidence for different structuring processes through time that ultimately led to a substantial change in the genetic constitution of the population over two centuries. We are aware that for the historical samples these indices are only rough estimates. They are based on small population samples and temporal sampling within periods. Nevertheless, individuals within samples were genetically strongly associated (high assignment probabilities) and scored consistently for diversity and variance estimates. For population Old, the relative indices high diversity (*H*_e_ = 0.68), negative inbreeding (*F*_is_ = −0.158), and low variance in heterozygote loci (0.52) indicate a relative large and stable population. The negative inbreeding estimate was not caused by sampling siblings, but rather evidences immigration into the population or results from differences in mating success of individuals throughout the sampled period. By contrast, in population Middle‐aged the relative indices were low diversity (*H*_e_ = 0.51), high inbreeding (*F*_is_ = 0.148), and intermediate variance in heterozygote loci (1.04). These *H*_e_ and *F*_is_ values clearly indicate a bottleneck in the population, which is also supported by our genetic drift simulations and the low estimated effective population size. Additionally, the variance in individual heterozygosity increases in the Middle‐aged population relative to the Old sample. Interestingly, this drift effect is directly seen at several loci (Table S5), where high‐frequency alleles increase and low‐frequency alleles become rarer or even disappear (Nei et al. 1975). The indices for the Young population are an intermediate diversity (*H*_e_ = 0.62), no inbreeding (*F*_is_ = 0.04), and a high variance in heterozygote loci (1.47). This sample differs from the two historical ones by being sampled at one point in time. Nevertheless, the rapid increase in diversity compared to the Middle‐aged population together with the by far highest variance in number of heterozygote loci suggests re‐immigration and an admixture of individuals with different pedigree ancestries. Because the studied loci are not linked and there is no evidence for selection on these loci, it is very unlikely that individuals of “different genetic quality” are responsible for the diversity differences among periods. Moreover, all sample material from population Old and Middle‐aged originated from trophies and thus represent high‐quality males despite a significant decrease in genetic diversity. Likewise, the increase in diversity between Middle‐aged samples (trophies) and Young (random samples) is contradictive to quality‐linked differences.

Because the mtDNA D‐Loop was monomorphic through time, immigration and mating success are most likely dominated by males. For population Old, the identification of a single haplotype was unexpected due to its large estimated effective population size. Compared to other geographical regions, where the D‐loop of red deer is highly variable (Ludt et al. 2004; Niedzialkowska et al. 2011), the haplotype in our study is new and indicates that the female population has been confined through time. Limited immigration by females as a consequence of strong philopatry, as seen in females (Fickel et al. 2012), could have restricted gene flow in the red deer of Neuwied. Limited immigration is common among many polygynous deer species (Nelson 1993; Pitra et al. 2004), as proven by both genetic and telemetry studies (Nussey et al. 2005; Skog et al. 2009). Lack of mtDNA sequence variation further provides evidence against translocations of females in the past decades. In other red deer populations with a history of translocation events, foreign haplotypes have been identified (Skog et al. 2009).

Gender‐biased gene flow is common in mammals (Kalz et al. 2006; Handley and Perrin 2007). Male‐biased gene flow is predicted by inbreeding avoidance theory as well as by local mate competition theory to occur in species with polygynous mating systems, such as in red deer (Cluttonbrock 1989; Perrin and Mazalov 1999, 2000). In highly mobile red deer, population reductions allow individuals from foreign populations to immigrate in order to search for mating opportunities and thereby introduce foreign alleles. That male red deer contribute more to the genetic diversity of offspring than females (Perez‐gonzalez et al. 2009) further demonstrates the immense impact hunting laws can have on red deer population structure. Males are coveted hunting prey, and the culling of dominant males may lead to immigration and reproduction success of individuals from other populations. Skewed mating success very quickly leads to a reduction in genetic variability, especially in areas without hunting laws, when few males remain after several rutting seasons. Also, the rigorous hunting schedules in the last decades, which protect individuals with large, branched antlers from harvesting (Hartl et al. 2003), could have imposed selection on particular males with small antlers prior to sexual maturity. This in combination with the polygynous mating system could also alter the frequencies of alleles within a few generations. The rapid changes observed in allele frequencies over a few generations in our study population highlight the endangerment of small populations exposed to overhunting and habitat fragmentation, but also that red deer populations are able to regenerate and increase population size and genetic diversity quickly within a few generations.

## Conflict of Interest

None declared.

## Supporting information


**Table S1.** List of individuals.
**Table S2.** Name, sequence, multiplex system, dilution, annealing temperatures and dye of used microsatellite primers.
**Table S3.** Primer sequences and annealing temperatures of specific forward and reverse primers for the amplification of the D‐Loop region between Pro‐tRNA and Phe‐tRNA of the mtDNA.
**Table S4.** Microsatellite genotypes of all 33 individuals in the three different periods Old (1813–1861), Middle‐aged (1923–1940) and Young (2011).
**Table S5.** Allele frequencies at 10 loci of population Old, Middle‐aged and Young.
**Appendix S1.** Study area and history.
