# Supplementary material for: Population dynamics of a natural red deer population over 200 years detected via substantial changes of genetic variation
Source: Ecol Evol. 2016 Apr 5;6(10):3146–53. doi: 10.1002/ece3.2063 (PMC4828584; doi:10.1002/ece3.2063)
Supplement: Supplementary file 1 — Table S1. List of individuals. Table S2. Name, sequence, multiplex system, dilution, annealing temperatures and dye of used microsatellite primers. Table S3. Primer sequences and annealing temperatures of specific forward and reverse primers for the amplification of the D‐Loop region between Pro‐tRNA and Phe‐tRNA of the mtDNA. Table S4. Microsatellite genotypes of all 33 individuals in the three different periods Old (1813–1861), Middle‐aged (1923–1940) and Young (2011). Table S5. Allele frequencies at 10 loci of population Old, Middle‐aged and Young. Appendix S1. Study area and history. [file ECE3-6-3146-s001.docx]

Table S1. List of individuals. Age refers to tissue age. Generation refers to the estimated past generation assuming a generation time of seven years.

| Individual | Population | DNA content | Age | Generation |
| --- | --- | --- | --- | --- |
| 1-17 | Young | nm | 2011 | 0 |
| 18 | Middle-aged | 400.5 | 1940 | -11.1 |
| 19 | Middle-aged | 224.7 | 1923 | -13.6 |
| 20 | Middle-aged | 66.2 | 1930 | -12.6 |
| 21 | Middle-aged | 182.6 | 1937 | -11.6 |
| 22 | Middle-aged | 180.7 | 1933 | -12.1 |
| 23 | Middle-aged | 119.8 | 1930 | -12.6 |
| 24 | Middle-aged | 123.7 | 1934 | -12.0 |
| 25 | Middle-aged | 240.6 | 1932 | -12.3 |
| 26 | Old | 67.1 | 1836 | -26.0 |
| 27 | Old | 178.8 | 1843 | -25.0 |
| 28 | Old | 306.3 | 1827 | -27.3 |
| 29 | Old | 93.0 | 1861 | -22.4 |
| 30 | Old | 123.0 | 1855 | -23.3 |
| 31 | Old | 76.1 | 1813 | -29.3 |

nm = not measured

Table S2: Name, sequence, multiplex system, dilution, annealing temperatures and dye of used microsatellite primers.

| **Locus** | **Primer Sequence (5’ - 3’)** | **System** | **Dilution** | **Annealing Temperature** | **Dye** |
| --- | --- | --- | --- | --- | --- |
| Haut14 | **F** ccagggaagatgaagtgacc  **R** tgaccttcactcatgttattaa | 2 | / | 56.0°C | Fam |
| ILSTS06 | **F** tgtctgtatttctgctgtgg  **R** acacggaagcgatctaaacg | 2 | / | 56.0°C | Ned |
| BM1818 | **F** agtgctttcaaggtccatgc  **R** agctgggaatataaccaaagg | 2 | 1:5 | 56.0°C | Hex |
| CSSM14 | **F** aaatgacctctcaatggaagcttg  **R** gaattctggcacttaataggattca | 2 | 1:3 | 56.0°C | Ned |
| CSPS115 | **F** aaagtgacacaacagcttctccag  **R** aacgagtgtcctagtttggctgtg | 2 | 1:2 | 56.0°C | Fam |
| CSSM16 | **F** agagccacttgttacaccccaaag  **R** gatgcagtctccacttgattcaaa | 1 | 1:5 | 56.0°C | Fam |
| MM12 | **F** caagacaggtgtttcaatct  **R** atcgactctggggatgatgt | 1 | 1:4 | 56.0°C | Hex |
| INRA35 | **F** ttgtgctttatgacactatccg  **R** atcctttgcagcctccacattc | 1 | 1:5 | 56.0°C | Ned |
| CSSM22 | **F** tctctctaatggagttggtttttg  **R** atatcccactgaggataagaattc | 1 | 1:3 | 56.0°C | Fam |
| CSSM19 | **F** ttgtcagcaacttcttgtatcttt  **R** tgttttaagccacccaattatttg | 1 | 1:3 | 56.0°C | Hex |

### Table S3: Primer sequences and annealing temperatures of specific forward and reverse primers for the amplification of the D-Loop region between Pro-tRNA and Phe-tRNA of the mtDNA.

| **Primer** | **Primer-Sequence (5’ – 3’)** | **Annealing Temperature** |
| --- | --- | --- |
| Forward | ACCCCCTGGAGTGCTAATTT | 59.9°C |
| F2 | TTTCATGAGTCAACCCTAAGATC | 57.4°C |
| F3 | GGATGCTTGGACTCAGCAAT | 60.2°C |
| F4 | TTTCAGGGCCATCTCACCTA | 60.5°C |
| F5 | TATCCCGTCCCCTAGATCAC | 58.9°C |
| Reverse | CAGCTTTCCACTCAACATCCA | 61.2°C |
| R1 | ATGGCAGTCAATGGTCACAG | 59.6°C |
| R2 | CTAATCAGCCCATGCTCACA | 59.8°C |
| R3 | CCATAAATTGTGGGGGTAGC | 59.2°C |
| R4 | GATGTTGTTCATCGTACATAG | 58.2°C |
| R5 | CATCCACTAACCACACAACAAAA | 59.8°C |

Table S4: Microsatellite genotypes of all 33 individuals in the three different periods Old (1813-1861), Middle-aged (1923-1940) and Young (2011).

| Locus | CSSM16 |  | MM12 |  | Inra35 |  | CSSM22 |  | CSSM19 |  |
| --- | --- | --- | --- | --- | --- | --- | --- | --- | --- | --- |
| Individual | Allel1 | Allel2 | Allel1 | Allel2 | Allel1 | Allel2 | Allel1 | Allel2 | Allel1 | Allel2 |
| Young1 | 154 | 162 | 84 | 84 | 101 | 107 | 208 | 212 | 142 | ? |
| Young2 | 156 | 162 | 84 | 86 | 101 | 117 | 212 | 212 | 150 | 160 |
| Young3 | 156 | 162 | 84 | 86 | 101 | 117 | 208 | 212 | 150 | 160 |
| Young4 | 154 | 162 | 84 | 86 | 107 | 111 | 208 | 212 | 142 | 160 |
| Young5 | 154 | 154 | 84 | 84 | 117 | 117 | 212 | 212 | 144 | 146 |
| Young6 | 154 | 162 | 84 | 84 | 111 | 117 | 208 | 212 | 146 | 160 |
| Young7 | 162 | 162 | 84 | 84 | 111 | 111 | 208 | 212 | 142 | 142 |
| Young8 | 154 | 162 | 84 | 84 | 111 | 117 | 212 | 212 | 146 | 150 |
| Young9 | 156 | 156 | 84 | 86 | 111 | 117 | 212 | 212 | 142 | 150 |
| Young10 | 154 | 154 | 84 | 84 | 99 | 115 | 208 | 212 | 150 | 150 |
| Young11 | 154 | 156 | 84 | 86 | 111 | 111 | 212 | 212 | 160 | 160 |
| Young12 | 154 | 162 | 84 | 84 | 99 | 109 | 208 | 212 | 150 | 160 |
| Young13 | 154 | 162 | 84 | 84 | 107 | 111 | 208 | 212 | 146 | 150 |
| Young14 | 162 | 162 | 84 | 84 | 99 | 115 | 212 | 212 | 142 | 146 |
| Young15 | 154 | 162 | 84 | 84 | 99 | 99 | 212 | 212 | 146 | 150 |
| Young16 | 156 | 156 | 84 | 84 | 111 | 117 | 212 | 214 | 142 | 150 |
| Young17 | 156 | 162 | 84 | 84 | 101 | 111 | 212 | 212 | 150 | 160 |
| Middle1 | 162 | 162 | 84 | 86 | 101 | 115 | 212 | 212 | 150 | 150 |
| Middle2 | 162 | 162 | 84 | 84 | 101 | 101 | 212 | 212 | 150 | 150 |
| Middle3 | 154 | 154 | 84 | 86 | 111 | 117 | 212 | 214 | 150 | 150 |
| Middle4 | 154 | 162 | 84 | 84 | 101 | 117 | 212 | 212 | 150 | 152 |
| Middle5 | 162 | 162 | 84 | 86 | 101 | 111 | 212 | 212 | 150 | 150 |
| Middle6 | 154 | 154 | 86 | 86 | 111 | 117 | 208 | 212 | 150 | 160 |
| Middle7 | 154 | 162 | 84 | 84 | 101 | 117 | 212 | 212 | 150 | 152 |
| Middle8 | 156 | 162 | 84 | 86 | 111 | 117 | 212 | 212 | 150 | 160 |
| Old1 | 156 | 162 | 84 | 84 | 101 | 107 | 212 | 212 | 146 | 150 |
| Old2 | 162 | 162 | 84 | 84 | 107 | 111 | 212 | 212 | 144 | 160 |
| Old3 | 156 | 162 | 84 | 84 | 107 | 107 | 212 | 214 | 150 | 160 |
| Old4 | 156 | 156 | 84 | 86 | 101 | 103 | 212 | 214 | 144 | 150 |
| Old5 | 156 | 164 | 84 | 86 | 101 | 107 | 212 | 212 | 144 | 150 |
| Old6 | 154 | 162 | 84 | 84 | 101 | 109 | 208 | 214 | 146 | 150 |

| Locus | Haut14 |  | CSPS115 |  | BM1818 |  | ILSTS06 |  | CSSM14 |  |
| --- | --- | --- | --- | --- | --- | --- | --- | --- | --- | --- |
| Individual | Allel1 | Allel2 | Allel1 | Allel2 | Allel1 | Allel2 | Allel1 | Allel2 | Allel1 | Allel2 |
| Young1 | 118 | 142 | 240 | 244 | 243 | 243 | 292 | 294 | 134 | 134 |
| Young2 | 130 | 142 | 244 | 244 | 237 | 243 | 296 | 300 | 134 | 134 |
| Young3 | 118 | 124 | 240 | 244 | 237 | 237 | 282 | 292 | 134 | 134 |
| Young4 | 130 | 142 | 240 | 244 | 243 | 245 | 292 | 294 | 134 | 134 |
| Young5 | ? | ? | ? | ? | 237 | 245 | 292 | 294 | 134 | 134 |
| Young6 | 116 | 118 | 238 | 244 | 247 | 247 | 294 | 296 | 134 | 136 |
| Young7 | 128 | 142 | 244 | 244 | 235 | 245 | 292 | 300 | 134 | 134 |
| Young8 | 116 | 118 | 238 | 238 | 247 | 247 | 292 | 300 | 134 | 134 |
| Young9 | 118 | 118 | 238 | 240 | 235 | 237 | 294 | 294 | 134 | 136 |
| Young10 | 118 | 142 | 240 | 244 | 243 | 247 | 280 | 294 | 134 | 134 |
| Young11 | 118 | 142 | 244 | 244 | 243 | 243 | 284 | 294 | 134 | 134 |
| Young12 | 104 | 142 | 244 | 244 | 235 | 243 | 280 | 284 | 134 | 134 |
| Young13 | 118 | 142 | 238 | 244 | 243 | 247 | 284 | 292 | 134 | 134 |
| Young14 | 122 | 142 | 244 | 244 | 237 | 243 | 288 | 300 | 134 | 134 |
| Young15 | 118 | 122 | 238 | 244 | 243 | 243 | 288 | 294 | 134 | 134 |
| Young16 | 118 | 118 | 240 | 244 | 235 | 237 | 284 | 294 | 134 | 134 |
| Young17 | 118 | 142 | 244 | 244 | 235 | 243 | 280 | 284 | 134 | 134 |
| Middle1 | 130 | 130 | 244 | 244 | 237 | 243 | 276 | 300 | 134 | 134 |
| Middle2 | 128 | 128 | 236 | 238 | 235 | 243 | 300 | 300 | 134 | 136 |
| Middle3 | 106 | 106 | ? | ? | 243 | 243 | 292 | 292 | 136 | 136 |
| Middle4 | 106 | 106 | 238 | 244 | 243 | 243 | 294 | 300 | 134 | 134 |
| Middle5 | ? | ? | ? | ? | 243 | 243 | 300 | 300 | 134 | 134 |
| Middle6 | 130 | 130 | 238 | 238 | 243 | 243 | 294 | 300 | 134 | 134 |
| Middle7 | 106 | 130 | 238 | 244 | 243 | 243 | 292 | 294 | 134 | 134 |
| Middle8 | 128 | ? | 244 | 244 | 243 | 243 | 292 | 300 | 134 | 134 |
| Old1 | 104 | 106 | 236 | 238 | 235 | 243 | 282 | 292 | 132 | 134 |
| Old2 | 106 | 108 | 244 | 246 | 243 | 245 | 276 | 294 | 132 | 134 |
| Old3 | 106 | 114 | 236 | 240 | 237 | 243 | 280 | 294 | 134 | 136 |
| Old4 | 104 | 118 | 238 | 244 | 235 | 245 | 276 | 290 | 134 | 134 |
| Old5 | 104 | ? | 236 | 240 | 243 | 247 | 276 | 276 | 134 | 134 |
| Old6 | 104 | ? | 236 | 244 | 243 | 245 | 292 | 294 | 134 | 134 |

Table S5. Allele frequencies at ten loci of population Old, Middle-aged and Young.

| **Locus 1** |  |  |  |  |  |  |  |  |  |  |  |
| --- | --- | --- | --- | --- | --- | --- | --- | --- | --- | --- | --- |
|  |  |  |  |  |  |  |  |  |  |  |  |
| Population | Alleles |  |  |  |  |  |  |  |  |  |  |
|  | 154 | 156 | 162 | 164 |  |  |  |  |  |  |  |
| Young | 0.353 | 0.235 | 0.412 | 0.000 |  |  |  |  |  |  |  |
| Middle-aged | 0.100 | 0.100 | 0.800 | 0.000 |  |  |  |  |  |  |  |
| Old | 0.100 | 0.300 | 0.500 | 0.100 |  |  |  |  |  |  |  |
|  |  |  |  |  |  |  |  |  |  |  |  |
| **Locus 2** |  |  |  |  |  |  |  |  |  |  |  |
|  |  |  |  |  |  |  |  |  |  |  |  |
| Population | Alleles |  |  |  |  |  |  |  |  |  |  |
|  | 84 | 86 |  |  |  |  |  |  |  |  |  |
| Young | 0.853 | 0.147 |  |  |  |  |  |  |  |  |  |
| Middle-aged | 0.625 | 0.375 |  |  |  |  |  |  |  |  |  |
| Old | 0.833 | 0.167 |  |  |  |  |  |  |  |  |  |
|  |  |  |  |  |  |  |  |  |  |  |  |
| **Locus 3** |  |  |  |  |  |  |  |  |  |  |  |
|  |  |  |  |  |  |  |  |  |  |  |  |
| Population | Alleles |  |  |  |  |  |  |  |  |  |  |
| Young | 0.147 | 0.118 | 0.000 | 0.088 | 0.029 | 0.324 | 0.059 | 0.235 |  |  |  |
| Middle-aged | 0.000 | 0.375 | 0.000 | 0.000 | 0.000 | 0.250 | 0.062 | 0.312 |  |  |  |
| Old | 0.000 | 0.333 | 0.083 | 0.417 | 0.083 | 0.083 | 0.000 | 0.000 |  |  |  |
|  |  |  |  |  |  |  |  |  |  |  |  |
| Locus 4 |  |  |  |  |  |  |  |  |  |  |  |
|  |  |  |  |  |  |  |  |  |  |  |  |
| Population | Alleles |  |  |  |  |  |  |  |  |  |  |
|  | 208 | 212 | 214 |  |  |  |  |  |  |  |  |
| Young | 0.235 | 0.735 | 0.029 |  |  |  |  |  |  |  |  |
| Middle-aged | 0.062 | 0.875 | 0.062 |  |  |  |  |  |  |  |  |
| Old | 0.083 | 0.667 | 0.250 |  |  |  |  |  |  |  |  |
|  |  |  |  |  |  |  |  |  |  |  |  |
| Locus 5 |  |  |  |  |  |  |  |  |  |  |  |
|  |  |  |  |  |  |  |  |  |  |  |  |
| Population | Alleles |  |  |  |  |  |  |  |  |  |  |
|  | 142 | 144 | 146 | 150 | 152 | 160 |  |  |  |  |  |
| Young | 0.188 | 0.031 | 0.188 | 0.344 | 0.000 | 0.250 |  |  |  |  |  |
| Middle-aged | 0.000 | 0.000 | 0.000 | 0.750 | 0.125 | 0.125 |  |  |  |  |  |
| Old | 0.000 | 0.250 | 0.167 | 0.417 | 0.000 | 0.167 |  |  |  |  |  |
|  |  |  |  |  |  |  |  |  |  |  |  |
| Locus 6 |  |  |  |  |  |  |  |  |  |  |  |
|  |  |  |  |  |  |  |  |  |  |  |  |
| Population | Alleles |  |  |  |  |  |  |  |  |  |  |
|  | 104 | 106 | 108 | 114 | 116 | 118 | 122 | 124 | 128 | 130 | 142 |
| Young | 0.031 | 0.000 | 0.000 | 0.000 | 0.062 | 0.406 | 0.062 | 0.031 | 0.031 | 0.062 | 0.312 |
| Middle-aged | 0.000 | 0.417 | 0.000 | 0.000 | 0.000 | 0.000 | 0.000 | 0.000 | 0.167 | 0.417 | 0.000 |
| Old | 0.250 | 0.375 | 0.125 | 0.125 | 0.000 | 0.125 | 0.000 | 0.000 | 0.000 | 0.000 | 0.000 |
|  |  |  |  |  |  |  |  |  |  |  |  |
| Locus 7 |  |  |  |  |  |  |  |  |  |  |  |
|  |  |  |  |  |  |  |  |  |  |  |  |
| Population | Alleles |  |  |  |  |  |  |  |  |  |  |
|  | 236 | 238 | 240 | 244 | 246 |  |  |  |  |  |  |
| Young | 0.000 | 0.188 | 0.188 | 0.625 | 0.000 |  |  |  |  |  |  |
| Middle-aged | 0.083 | 0.417 | 0.000 | 0.500 | 0.000 |  |  |  |  |  |  |
| Old | 0.333 | 0.167 | 0.167 | 0.250 | 0.083 |  |  |  |  |  |  |
|  |  |  |  |  |  |  |  |  |  |  |  |
| Locus 8 |  |  |  |  |  |  |  |  |  |  |  |
|  |  |  |  |  |  |  |  |  |  |  |  |
| Population | Alleles |  |  |  |  |  |  |  |  |  |  |
|  | 235 | 237 | 243 | 245 | 247 |  |  |  |  |  |  |
| Young | 0.147 | 0.206 | 0.382 | 0.088 | 0.176 |  |  |  |  |  |  |
| Middle-aged | 0.062 | 0.062 | 0.875 | 0.000 | 0.000 |  |  |  |  |  |  |
| Old | 0.167 | 0.083 | 0.417 | 0.250 | 0.083 |  |  |  |  |  |  |
|  |  |  |  |  |  |  |  |  |  |  |  |
| Locus 9 |  |  |  |  |  |  |  |  |  |  |  |
|  |  |  |  |  |  |  |  |  |  |  |  |
| Population | Alleles |  |  |  |  |  |  |  |  |  |  |
|  | 276 | 280 | 282 | 284 | 288 | 290 | 292 | 294 | 296 | 300 |  |
| Young | 0.000 | 0.088 | 0.029 | 0.147 | 0.059 | 0.000 | 0.206 | 0.294 | 0.059 | 0.118 |  |
| Middle-aged | 0.062 | 0.000 | 0.000 | 0.000 | 0.000 | 0.000 | 0.250 | 0.188 | 0.000 | 0.500 |  |
| Old | 0.333 | 0.083 | 0.083 | 0.000 | 0.000 | 0.083 | 0.167 | 0.250 | 0.000 | 0.000 |  |
|  |  |  |  |  |  |  |  |  |  |  |  |
| Locus 10 |  |  |  |  |  |  |  |  |  |  |  |
|  |  |  |  |  |  |  |  |  |  |  |  |
| Population | Alleles |  |  |  |  |  |  |  |  |  |  |
|  | 132 | 134 | 136 |  |  |  |  |  |  |  |  |
| Young | 0.000 | 0.941 | 0.059 |  |  |  |  |  |  |  |  |
| Middle-aged | 0.000 | 0.812 | 0.188 |  |  |  |  |  |  |  |  |
| Old | 0.167 | 0.750 | 0.083 |  |  |  |  |  |  |  |  |

Appendix S1: Study area and history

The area is located at the westernmost edge of a nature preserve (“Rhein Westerwald”). The landscape is typical for the river Rhine; land use alternates between vineyards and steep forested hills. About 45 % of the area is forested while *c*. 34 % is used for agriculture. The 10 km^2^ area, from where samples came from, belongs to the private estate of the princes of Neuwied who were the only people hunting and managing red deer there. In Germany, hunting is area based, not license based as in many other parts of the world. The 10 km^2^ is part of a larger area that has never been completely fenced during the last centuries, so gene flow with other populations was not restricted.

In the period 1813-1861 the Neuwied population was large. There were few barriers to migration and there was a low degree of fragmentation in an overall continuous habitat. Prior to 1848 only nobles were allowed to hunt. They managed the red deer populations and deer were only harvested in a few hunts per year. The most popular hunting items were older males with large antlers. During the revolution years 1848-1849 there were no hunting laws. This period saw massive reductions and large-scale extinctions in red deer populations nation-wide.. During the second half of the 19^th^ century, hunting laws were again put into action which allowed populations to re-establish in many areas (Kuehn *et al.* 2003). After re-establishment, population sizes were once again reduced as a response to increasing damages to agriculture, but also caused by poaching during and after World War 1. Our second population samples come from the period representing the end-phase of overexploitation (1923-1940). Following the 1950’s, red deer populations expanded into many forested areas. For the first time, rigorous hunting schedules favoring large, branched antlers were introduced (Hartl *et al.* 2003). Young males with small antlers were harvested while males with large antlers were allowed to reach maturity and reproduce. Middle-aged to old males up to 10 years with large antlers were normally not harvested. The contemporary Neuwied population is more fragmented than in former times. It is confined to the south by the river Rhine, and in the west and east it is fenced by motorways and highways. Migration and gene flow is presumably highly restricted in this population. Translocation events into the continuous population are not known. We do not know the population sizes of red deer during the three time periods except that the population was nearly eradicated during and after the revolution in 1848.
